# Ultrasonographic Elasticity Contrast Index of Palpable Breast Lumps

**DOI:** 10.31729/jnma.3536

**Published:** 2018-08-31

**Authors:** Anamika Jha

**Affiliations:** 1 Department of Radiology & Imaging, Institute of Medicine, Tribhuvan University Teaching Hospital, Maharajgunj, Kathmandu, Nepal

**Keywords:** *Breast cancer*, *Elastography*, *Strain Elastography*, *Ultrasonography*

## Abstract

**Introduction:**

Elastrography used in addition to grey scale sonography increases its specificity. Elasticity contrast index (ECI) is based on strain elastrography and being a quantitative parameter, maybe more easy to obtain and reproducible while researches has been done in ECI in thyroid lesions, this is the first study, to the best of our knowledge to evaluate in breast lesions. This study was done to evaluate the diagnostic accuracy of Elasticity Contrast Index (ECI) in differentiating benign from malignant lesions of breast and to determine its cutoff value.

**Methods:**

This is a descriptive cross-sectional study done at tertiary health care centre, which involved retrospective evaluation of data collected from September 2016 to March 2017. Conventional sonography was done followed by elastography on commercially available ultrasound machine. ECI was calculated in thyroid protocol available in the unit. Histopathological diagnosis was obtained for all the lesions and taken as gold standard.

**Results:**

A total of 89 breast lumps were evaluated, of which was 61 (69.3%) were benign and 27 (30.7%) malignant on histopathology. Independent t test revealed the average ECI value of benign lesions was 2.48 and malignant 5.1. Receiver operating curve showed ECI value of 3.25 as the cutoff, above which the lesions were malignant.

**Conclusions:**

ECI is a quantitative elastography technique which can be easily used as an adjunct during breast sonography and can increase its specificity for diagnosing a lesion as malignant. This could reduce the number of false positive biopsies.

## INTRODUCTION

The specificity of grey scale sonography can be increased by adding elastographic techniques to the conventional technique, thus, limiting aspiration biopsies to the more suspicious lesions.^1^ Most of the previously performed studies are based on qualitative and semi-quantitative elastographic evaluation using strain score or ratio which may have greater inter and intra-observer variability. Shear-wave elastography is a quantitative and reproducible technique but is limited by its availability on the commercial scanners.^2,3^

Elasticity Contrast Index (ECI) based on strain elastography, is a quantitative technique, easy to obtain and reproducible. To the best of our knowledge, there is no published literature regarding the ECI of breast lesions. So, in this study we evaluate the diagnostic accuracy of ECI in evaluating benign and malignant breast lesions and determine its cutoff value for malignant lesion.

## METHODS

This descriptive cross-sectional study involved retrospective evaluation of data collected from September 2016 to March 2017 after obtaining ethical approval from the institutional review board. Non-probability convenience sampling was used.

The patients who underwent breast sonography with sonographically evident palpable breast lumps and whose histopathological diagnosis could be obtained, were included in the study. Typical simple cysts were excluded from the study. A total of 89 individuals (88 Females and 1 Male) of different age groups were evaluated, of which 1 patient later refused to participate and had to be excluded.

Grey scale evaluation was performed using high frequency (7–12 MHz) probe (MEDISON ACCUVIX A30). The quantitative strain elastography of the breast lesions was performed with thyroid protocol in which it was available. To obtain the ECI value, ROI was placed to include the largest solid part of the lesion. ECI was displayed on the monitor. Minimum two measurements were obtained for each lesion and the lowest values were recorded. Sonographic categorization of lesions into various BIRADS (breast imaging reporting and data system) categories was done as per the ACR protocol.^4^ BIRADS 2 and3 lesions were considered benign, 4a and 4b indeterminate and 4c, 5 and 6 malignant. Histopathological diagnoses of all lesions were obtained, which was taken as the gold standard.

Indeterminate lesions were further categorized according to the final histopathological diagnosis as benign nonspecific, benign neoplastic like fibroadenomas and phylloides tumor, benign intraductal papilloma and inflammatory lesions and malignant.

SPSS 16 was used for statistical analysis. Receiver operating characteristic curve was prepared to evaluate the diagnostic accuracy of ECI for differentiating the benign from malignant lesions. Cutoff value of ECI for malignant lesion was determined. Independent t-test was used to compare the ECI value of benign and malignant solid lesions.

## RESULTS

The study group included 87 females and 1 male with age ranging from 17–86 years. The mean age in benign group was 32.6 and that in malignant was 49 years. The mean size of lesion in benign group was 2.5 x 1.6 cm and in malignant was 3.0 x 2.0 cm. The minimum size of the lesion in this study was 0.8 x 0.6 cm. Independent t-test showed the ECI of 5.1 for malignant and 2.48 for the benign lesions, with p value less than 0.001.

**Figure 1.**
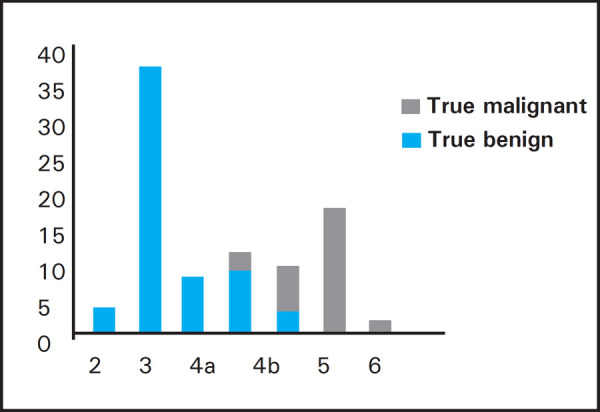
BIRADS category distribution of the lesions

Sonographically, there were 32% malignant, 21 % indeterminate and 47% benign lesions and on histopathology 30.7% were malignant and 60.3% benign (Figure 1).

**Table 1:** ECI value of indeterminate lesions.

Category	Average ECI Value
Benign (n = 2)	1.3
Benign mass like (n=4)	3.8
Benign IDP (n=4)	3.7
Inflammatory (n = 6)	3.4
Malignant (n=2)	4.7

Among 28 sonographically malignant masses, 3 had low ECI values of 2.3 (periductal mastitis), 2.6 (intraductal papilloma) and 2.5 (foreign body granuloma) and were histopathologically benign. Among the indeterminate lesions, histopathologically proven malignant ones had a higher average ECI (4.7) as compared to benign which ranged from 1.3–3.8 (Table 1).

**Figure 2.**
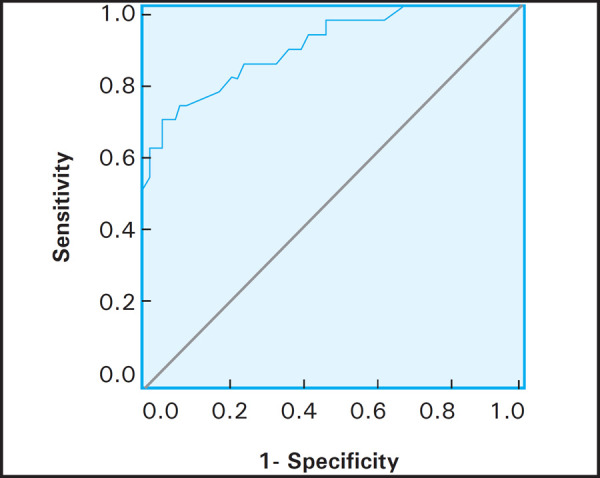
ROC curve.

ROC curve revealed the area under the curve is 0.896 (95% confidence interval), (0.823–0.97) and ECI cutoff value of 3.25 (Figure 2). ECI showed sensitivity of 85.2, specificity 73.3, positive predictive value of 59, and negative predictive value of 91.7 for diagnosing a malignant lesion.

## DISCUSSION

Palpation is the subjective traditional clinical way of assessing the stiffness of a lesion and elastography is the objective technique for the same. Elasticity of a lesion is described as qualitative estimation of tissue's Young modulus which is the ratio of external compression ‘stress’ and the deformation of the tissue because of this compression i.e. strain.^5,6^ The two elastography techniques currently in practice are strain and shear wave elastography which involve manual compression of the mass or transmission of ultrasonic energy into the mass, respectively.^7^

Grey scale sonography has high sensitivity of more than 94% for malignant breast lesions, but is limited by a relatively low specificity varying from 40–83%.^8^ This leads to higher false positives and increased number of image guided biopsies with low cancer detection rate varying from about10–30%.^1,9,10^ Various studies have shown that adding elastography to routine grey scale imaging in breast lesions increases the specificity to 85–90 %.^5,8^

Qualitative elastography has also been included in the current ACR BIRADS lexicon 2013 as an ancillary finding and the descriptors for elasticity assessment included. Various qualitative and semiquantitative elastography techniques studied have shown reasonable diagnostic accuracy in differentiating benign and malignant breast masses.^11,12^

Some elastographic techniques in use are Tsukuba score, strain ratio and Elasticity Index (EI)/B-mode ratio. Tsukuba score, a five-point scoring system based on color of the lesion, is the first classification system for elastography in literature. With increasing Tsukuba score, there is higher probability of lesion being malignant.^13^ This scoring system has been validated by other studies and found to be complimentary to grey scale sonography, especially for the BIRADS 3 and 4 lesions.^14,15^

Strain ratio or Fat-Lesion Ratio (FLR) as defined by Ueno et al. is the ratio of the mean strain of fat by the mean strain of solid lesion. Above a ratio of 4.8, the lesion is more likely to be malignant.^16^

A semi-quantitative elastographic technique described by Barr RG et al. is EI/B-mode ratio, where the lesion size measured on the elastogram is divided by that on the B-mode sonography. At EI/B-mode ratio cut-off of > 1.0 lesion was more likely to be malignant.^17^

Comparative studies found the semiqualitative elasticity ratio to be superior to elasticity score in assessing tissue stiffness.^18^ Another study comparing the EI with the elasticity ratio (ER), latter obtained as the ratio of EI of the lesion and EI of the reference, suggested that EI may solely be used for evaluation of soft tissues.^19^

We also found ECI (same as EI) to have a high diagnostic accuracy, to be easily reproducible and more objective. We suggest that ECI, a quantitative elastography parameter, must be utilized for further characterization of the lesion in addition to conventional sonography, whenever available in the ultrasonography unit.

Our study is limited by the unavoidable difference in the amount of manual compression applied which may lead to inter and intraoperator variability in obtaining the ECI values, with higher stiffness values at greater compression. We tried to limit this by keeping the probe at the surface applying very light pressure. Also, the ROI may be limited for very small lesions which needs to be evaluated further. There is further scope for evaluating this ECI cut-off value in a larger sample size and reviewing the diagnostic accuracy. More comparative studies with other elastography score, ratio and indices also need to be done.

## CONCLUSIONS

This study concludes that ECI has high diagnostic accuracy in prediction of breast malignancy and above a cut-off value, lesion is more likely to be malignant. It can be a helpful adjunct to routine grey scale sonography increasing its specificity and reducing the number of false positive biopsies.

## Conflict of Interest

**None.**
